# Disease-emergence dynamics and control in a socially-structured wildlife species

**DOI:** 10.1038/srep25150

**Published:** 2016-04-26

**Authors:** Kim M. Pepin, Kurt C. VerCauteren

**Affiliations:** 1 National Wildlife Research Center, United States Department of Agriculture, Animal and Plant Health Inspection Service, Wildlife Services, 4101 Laporte Ave., Fort Collins, CO, 80521.

## Abstract

Once a pathogen is introduced in a population, key factors governing rate of spread include contact structure, supply of susceptible individuals and pathogen life-history. We examined the interplay of these factors on emergence dynamics and efficacy of disease prevention and response. We contrasted transmission dynamics of livestock viruses with different life-histories in hypothetical populations of feral swine with different contact structures (homogenous, metapopulation, spatial and network). Persistence probability was near 0 for the FMDV-like case under a wide range of parameter values and contact structures, while persistence was probable for the CSFV-like case. There were no sets of conditions where the FMDV-like pathogen persisted in every stochastic simulation. Even when population growth rates were up to 300% annually, the FMDV-like pathogen persisted in <25% of simulations regardless of transmission probabilities and contact structure. For networks and spatial contact structure, persistence probability of the FMDV-like pathogen was always <10%. Because of its low persistence probability, even very early response to the FMDV-like pathogen in feral swine was unwarranted while response to the CSFV-like pathogen was generally effective. When pre-emergence culling of feral swine caused population declines, it was effective at decreasing outbreak size of both diseases by ≥80%.

Contact is a fundamental component of pathogen transmission for any directly transmitted pathogen. There are two main components of contact: rates or probabilities that individuals contact one another, and structure – the way a population mixes defining which individuals contact one another. Deviations from a homogenously mixing population constitute contact heterogeneities which can affect outbreak probability, size and progression^1^,^2^,^3^. In wildlife species, contact heterogeneities can arise due to social structure, fluctuating demographic dynamics, landscape or other ecological aspects of the population^4^; affecting disease spread rates and extinction probabilities. Much of what we know about the effects of contact heterogeneities on disease transmission have been derived from models of human or livestock systems (e.g.^5^,^6^,^7^) which can vary tremendously in both contact structure and demographic dynamics from populations of free-ranging wildlife.

Recent field studies in wildlife systems have revealed heterogeneous contact structures, providing foundational quantities for informing contact ecology^8^,^9^,^10^,^11^,^12^,^13^,^14^, and demonstrating major implications for disease management^15^,^16^. In addition, contact structure can limit outbreak size and outbreak probability more in diseases with shorter infectious periods^9^, emphasizing the importance of understanding the interplay of contact structure, demographics and disease life-history for determining optimal disease management strategies.

Wild pigs (*Sus scrofa*; often termed feral swine in the USA) are an example of a social wildlife species, which occur on most continents globally. Similar to other social wildlife species, feral swine occur in family groups. Individuals within groups are thought to interact more with each other than with individuals from other groups^17^. Family group structure is dynamic in that males disperse at reproductive maturity, females disperse to form new groups when groups become too large and new births occur throughout the year (Fig. 1). This inherently leads to heterogeneous contact structure^13^,^18^.

In Europe, wild pigs have been implicated in the maintenance and spillover of economically devastating livestock pathogens such as classical swine fever virus (CSFV^19^,^20^). In contrast, evidence for implicating wild pigs in spillover of other important livestock pathogens such as foot-and-mouth disease virus (FMDV) is weak^19^,^21^, even though wild pigs can be productive hosts of FMDV^22^. Previous studies in Europe and Australia suggest that social structure likely plays a key role in determining the ability of wild pigs to maintain pathogens such as FMDV^20^,^23^,^24^. For example, a modeling study based on feral swine and white-tailed deer distributions in southern Texas, USA^25^ found that FMDV could persist in white-tailed deer populations due to the contiguity of their distribution, while persistence was much less certain in feral swine due to their more heterogeneous distribution. Similarly, in wild pig distributions in Australia, FMDV did not persist when transmission was limited to wild pig populations^26^, while CSFV was able to persist and spread like a wave across the landscape^27^. Given these findings and previous work on the interplay between infectious period and contact structure^9^, we hypothesize that pathogens with short infectious periods such as FMDV may not persist in USA feral swine compared with a disease such as CSFV with a longer infectious period.

Feral swine occur broadly across southern USA^28^ near livestock facilities posing a potential threat to the emergence of livestock diseases such as FMD and CSF, which are currently not present in the USA. To maximize effectiveness of wildlife disease management resources and contingency plans, an understanding of management actions based on realistic contact ecology and disease-specific life-history is needed. In a recent study, we compiled GPS data from feral swine across southern USA to gain a better understanding of contact ecology in USA landscapes for the purpose of risk assessment^13^. Guided by results of this previous study, here we assessed the dynamics of two pathogens with different incubation and infectious periods under five different assumptions about social structure (Fig. 1) to better understand how contact structure determines emergence dynamics depending on pathogen life-history.

We focused on incubation and infectious periods that have been observed for FMD and CSF viruses because they are economically important pathogens with substantially different life histories. Throughout the text we refer to these viruses as FMDV-like and CSFV-like because we make simplifying assumptions (described in the Methods) about some biological aspects of these pathogens. We examined emergence dynamics under a range of population growth rates to mimic feral swine populations in the USA that are growing or declining due to management intensity, and in order for our results to have relevance to other social host species (e.g., white-tailed deer which are widely distributed, social and include a wide range of population abundance an growth rates). Lastly, we investigated the efficacy of pre-emergence management and post-emergence response actions to better understand the importance of contact structure for planning culling of feral swine populations.

## Results

### Outcome of disease dynamics

As expected, metrics of disease transmission depended on the interplay of contact structure and disease life-history (Fig. 2). For example, the CSFV-like virus almost always persisted until the end of simulations (2.5 years) for all contact structures except networks, whereas the FMDV-like virus only persisted occasionally in contact structures with the most homogenous mixing (homogenous or metapopulation) and rarely under spatial or network structures (Fig. 2). Also, networks (mean degree 3 or 6) limited total cases, spatial spread and outbreak probability proportionately more severely for the FMDV-like virus than the CSFV-like virus relative to other contact structures. R_0_ for both diseases was very limited by spatial and network contact structures.

### Sensitivity analyses

The spatial and network contact structures severely limited persistence probability and total cases for both diseases (Fig. 3). Transmission rate and conception probability generally had the strongest impact on outbreak metrics (Table S1). In the spatial models, initial density and dispersal distance also had strong impacts on outbreak metrics (Table S1). In fact dispersal distance and initial density had a stronger impact on total cases than conception probability in spatial models of the CSFV-like virus (Table S1). Strikingly, persistence probability was near 0 for the FMDV-like virus under a wide range of parameter values and contact structures. Relative to within-group transmission rates, between-group transmission rates had a smaller impact on total cases in the contact structures that included family group structure (Fig. 3B).

Our model showed a population growth rate threshold effect on persistence probability and mean total cases (Fig. 4). For the FMDV-like virus to persist in 10% of simulations, the contact structure had to be homogenous between groups of feral swine and the population growth rate had to be >85% annually (212% over the 2.5 years following introduction) which corresponded to an initial population size of ~1000 at the time the disease was introduced (Fig. 4A). However, if the effective population size for transmission was reduced by spatial or network-based contact structure, the FMDV-like virus almost never persisted even when annual population growth rates were ~300% which corresponded to an initial population size of ~1300 individuals. In contrast, the CSFV-like virus managed to persist at an annual growth rate of ~15% (initial population size of ~500 at the time of disease introduction), and was able to persist even in spatial and network contact structures. For simulations where neither virus persisted to the end, average persistence time was always well under 100 days for the FMDV-like virus while it was close to 300 days for the CSFV-like virus (Fig. 4B). The proportion of simulations that experienced outbreaks (i.e. >10 cases) were relatively unaffected by population growth rate, but higher when between-group contact structure was homogenous (Fig. 4C). For simulations that experienced an outbreak, mean total cases increased dramatically beyond annual population growth rates of 85% (initial population size of ~1000) for both diseases (Fig. 4D). However, for the FMDV-like virus, total cases were more strongly limited in spatial and network-based contact structures, relative to the CSFV-like virus.

As expected, the probability of persistence and mean total cases were negatively related to dispersal distance in the spatial contact structures (Fig. 5), although the strength of these effects differed between the two diseases. For the FMDV-like virus, persistence and total cases were very low once dispersal distance was twice (at 4 km) the radius of the between-group contact zone (2 km). However, for the CSFV-like virus persistence and total cases were still significant at 5 times (at 10 km) the radius of the contact zone.

### Pre-outbreak population management

We examined the effects of regular population management actions by culling fixed numbers of individuals at a fixed time interval. Culling activities began one year prior to disease introduction, and ranged from culling ~1–35% of the population during the first culling event. Considering an average effect over all sets of conditions, management was similarly effective for both diseases overall but made more of a difference at lower culling rates for the CSFV-like virus (20% disease-induced mortality) relative to the FMDV-like virus or the CSFV-like virus (80% disease-induced mortality) (Fig. 6). Another difference was that the average uncertainty associated with the effects of management was higher for the FMDV-like virus and network contact structures (Fig. 6, S4), likely due to the lower total cases and outbreak probability.

Management was less effective overall when culled individuals were removed randomly across the landscape (Figures S5 and S6) versus when each culling event involved neighboring individuals (Fig. 6, top) or high-degree network nodes and their neighbors (Fig. 6, bottom). For both diseases and a host population size of 575 at the outset of culling, removing 10 individuals (~1.2% of population during first event) every 10 days for 1 year prior to disease introduction was needed in order to reduce cases >80% (Fig. 6). These culling parameters equate to a net population decline of 66 ± 26% annually prior to introduction of the pathogen. Other management scenarios produced similar results (e.g., removal of 100 feral swine every 90 days which equates to a rate of population decline of 59 ± 26% annually). In contrast, 60% case reduction could be achieved with levels of management equating to population growth rates near 0 (e.g., 50 feral swine every 60 days: −5.1 ± 10% annual population growth rate). Also, for the CSFV-like virus with only 20% mortality, 60% case reduction could be achieved by decreasing population growth rate from 42 ± 2.5% to 16 ± 6.5% (i.e., while maintaining a net positive growth rate).

### Post-outbreak response

In order to examine efficacy of response to disease emergence, we simulated the effects of different culling intensities following the introduction of disease. We assumed efforts would occur daily beginning at the time a positive case was identified. We also assumed that culling would be targeted to individuals in spatial proximity (spatial metapopulations) or connected to (networks) infectious individuals. Striking results are that even with unrealistically early detection and high-intensity culling, response is relatively ineffective for reducing cases of the FMDV-like virus (Fig. 7). Oppositely, for the CSFV-like virus, culling at any intensity is highly effective at minimizing cases, although the efficacy decreases with higher DIM (Fig. 7). Again, uncertainty is quite high for the FMDV-like virus where stochastic fade out is more common, relative to the CSFV-like virus (Figure S7) which was more persistent (Figs 2, 3, 4).

## Discussion

We previously analyzed GPS data collected from wild pigs throughout southern USA and found that contact structure was highly heterogeneous^13^. In particular, contact between groups with home range centroids >2 km apart was rare, and social networks were found to be highly redundant with low connectedness. In the current study we incorporated this spatial and network information to examine the emergence dynamics of two viral diseases with different life-histories. We showed that under realistic demographics and contact structure, an FMDV-like disease would fade out quickly in feral swine whereas a CSFV-like disease could persist for long periods causing many more total cases. These results are consistent with observations of FMDV and CSFV antibody dynamics in wild pigs in Europe^29^,^30^,^31^ as well as a qualitative assessment of the risk of persistence of these diseases in pigs^19^.

Contact within wildlife populations is usually heterogeneous^4^ due to behavioral and/or landscape characteristics. Our simulations showed how the combined effects of disease life-history and realistic variations of contact structure can largely impact the probability and severity of disease emergence, emphasizing the need to decrease uncertainty in our understanding of contact structure for risk assessment purposes. Aside from contact structure, absolute transmission rates and population growth rates were generally the most important parameters for predicting the number of cases that will occur following an introduction event. Because transmission rate is proportional to contact rate in a directly-transmitted disease, absolute measures of contact rate would be very informative for risk assessment. Carefully designed studies that directly measure contact (rates and structure) and host demographics will help with practical quantification of risk. Conducting these studies in areas near livestock will provide the best assessment of disease spillover risk from feral swine.

For the management and response simulations, pathogens were introduced in a population size ~1000 (which corresponded to conditions with 85% growth rate annually). We did this because we hypothesized that contiguously interacting feral swine populations (i.e. boundaries of contact on a time scale relevant to disease transmission) in the USA are fragmented into small populations due to landscape and population reduction activities (e.g., hunting and culling by wildlife population managers). However, there are some areas in the USA that may have large contiguous populations, for example, in Texas. It is likely that persistence of FMDV-like viruses could be increased in these larger populations (e.g.^25^) and thus our results should not be extrapolated to large contiguous populations. Rather, our results show emergence dynamics in a range of smaller populations (specific size controlled through population growth rate), which can be used to guide pre-emergence management in space. For example, if a case is found in a large contiguous population, the best culling strategy may include culling feral swine in a ring around the area where the case was identified, limiting the population size within the ring to <1000 individuals. Population size estimates and contact structure across areas with suspected large contiguous populations, would further help in implementing this type of management plan in a way that minimizes disease emergence. Similarly, understanding typical population sizes for isolated populations and migration rates between them could be important for understanding risk of emergence and predicting spatial spread.

Regular population reduction activities conducted before emergence were effective at decreasing the total number of cases. However, culling activities needed to cause a population decline of ~15% in order to reduce outbreak size by ~80%. The obvious importance of high-intensity, regular culling for preventing emergence suggests that prevention strategies that focus on local host eradication will perform best. Because the FMDV-like virus faded out quickly in the absence of culling under network contact structures, weak culling intensity had low and uncertain effects on preventing emergence under these conditions, which supports previous simulation modeling work^26^.

Similarly, response to the FMDV-like virus was ineffective unless the response was initiated within a few days of introduction. In contrast, response to the CSFV-like virus was highly effective even if it was not detected until 60 days after the index case, which is consistent with results from a trapping campaign in Bulgaria^32^ and a simulation model depicting Australian conditions^27^. Our results suggest that allotting resources for intense culling of feral swine *after* an FMDV-like virus has emerged may be relatively fruitless because the virus will likely be self-limiting whereas culling in response to a CSFV-like virus could be highly effective. The result that response to the FMDV-like virus via culling feral swine would be inefficient has also been observed in a previous model based on an Australian scenario that included spillover to cattle^26^. Research to elucidate actual contact structures will allow for validation of these conclusions.

High disease-induced mortality decreased efficiency of response to the CSFV-like virus in spatial contact structures but not networks. This was because disease invasion occurred very quickly early on in the spatial metapopulation contact structures but much slower in the networks. Thus, hosts began to die on their own due to disease by the time response was initiated in the spatial metapopulation structure whereas disease-induced mortality had not yet had much of an impact on transmission in the networks. This result highlights that: 1) accurate knowledge of contact structure is important in planning resource allocation and response strategies, and 2) differences in contact structure and disease-induced mortality could account for some of the mixed outcomes of previous control efforts in other countries^33^.

Although we used results from a previous study^13^ to guide the structure and parameters of the contact structures we used in the present study, the empirical data are only a rough estimate. In the previous study, contact structure parameters were quantified from a metaanalysis of data that were collected during studies with goals other than measuring contact, suggesting there are likely missing data (i.e., the sampling design did not include all individuals within a given spatial area). Nonetheless, the previous work found that networks from three studies (including data from TX and FL) all had high transitivity, even when indirect contacts were included. Furthermore, the average degree was low (1.2–3.4) for direct between-group contact. For networks of indirect contact (up to 1 month between visits to the same environmental feature), average degree ranged from 2.8–7.4. Thus, in the current study, we also investigated a network with degree 6 to account for missing data and the potential for indirect contact (which is known to occur for CSFV^20^,^34^ and possibly FMDV^21^,^35^). However, the network degree had very little impact on most outbreak metrics except that the probability of R_0_ = 0 was ~10% lower for the FMDV-like virus transmission on the network with mean degree =6. Overall, network structure for both degrees (3 and 6) did result in lower outbreak metrics relative to other contact structures. This suggests that differences in network degree within this relatively low range of degree space has little impact on most outbreak metrics.

In contrast, for transmission in spatial contact structures, we found that properties mattered: mean dispersal distances within the spatial radius of between-group contact structure dramatically increased outbreak size and affected outbreak probability. Taken together, our results suggest that joint consideration of network properties, spatial contact and dispersal distances may be the most important aspects of feral swine contact structure for determining outbreak metrics. Field studies designed to quantify these properties absolutely will provide more accurate and tangible risk assessment.

Another caveat of our analyses was that our model of virus transmission was relatively simple compared with the actual biology of CSFV and FMDV. It is known that CSFV can be transmitted from mother to offspring during gestation and that this route of transmission can lead to persistent infection followed by death^20^,^34^. The infection can also be acute (lasting under 10 days) or chronic (lasting >1 month)^20^,^34^. The relative proportions of these conditions and their associated levels of disease-induced mortality depend on strain and condition of the host. We assumed a Poisson distribution of infectious periods with no mother-to-offspring transmission, and used a range of disease-induced mortality. Our approach accounts for the wide variation in infectious period due to different manifestations of the disease without representing the different mechanisms explicitly, but does not account for the additional cases that could be potentially produced through mechanisms such as vertical transmission. Thus, our estimates of transmission and persistence probability of the CSFV-like virus could be underestimates for CSFV.

With regards to FMDV, we ignored disease-induced mortality which can be significant especially in juveniles^36^. We also ignored behavioral responses in host movement due to disease-induced morbidity. Both of these simplifications lead to more disease transmission than would occur if the disease-induced effects of FMDV were accounted for, which makes our approach for examining persistence ability in FMDV-like viruses conservative. That is, we observed low persistence of FMDV-like viruses despite allowing ample transmission opportunity given other aspects of FMDV life-history. Lastly, we did not address strain-specific variation in incubation and infectious periods for FMDV-like viruses because we used a single value for the mean infectious period (7 days). However, we did account for individual variation (which can occur due to dose, route of exposure or host physiological status) by using Poisson-distributed random numbers for infectious periods. The range we captured using this approach to modeling infectious periods (1–16 day infectious periods with 5–9 days occurring most often) is similar to that observed in an experimental comparison of FMDV strains with different virulence levels in pigs^37^. Based on our results, longer mean infectious periods potentially could lead to higher persistence probability (as seen for the CSFV-like case) and shorter infectious periods would have the opposite effect.

One last caveat is that other wildlife or livestock can play an important role in pathogen dynamics in feral swine^21^,^26^. An Australian study^26^ that considered pig-cattle transmission based on empirical distributions of wild pigs and cattle, showed that FMDV persisted in pig populations when spillback from cattle occurred but did not persist when transmission was among pigs alone. Furthermore, a similar study based on feral swine and white-tailed deer distributions in southern Texas, USA^25^, which modeled persistence of FMDV separately in each species, found that FMDV could persist in white-tailed deer quite well. If contact structure of feral swine involves frequent interaction with other species that transmit and maintain FMDV, for example, multiple spillover-spillback events could possibly enable persistence. A field survey of contact among species that are susceptible to target pathogens, combined with simulations would help to elucidate the risk of pathogen persistence given a multi-host species context.

In conclusion, feral swine in the USA are likely to be a poor reservoir species for a FMDV-like virus, whereas the opposite may be true for a CSFV-like virus. Pre-emergence routine culling can be very effective at minimizing the risk of emergence of both pathogens, but these activities need to lead to net population declines for ≥80% effectiveness. Post-emergence response to an FMDV-like virus is predicted to be highly inefficient because of the high probability of natural fade out. In contrast, targeted response to a CSFV-like virus can be highly effective even when the disease is not identified until 60 days after introduction, but the level of effectiveness depends on contact structure. Field studies designed to measure contact structure in different settings are key to providing practical estimates of risk assessment.

## Methods

### Model overview

We used a spatially-explicit agent-based model of population dynamics and disease transmission implemented in Matlab (Version R2015a, The MathWorks, Inc., Natick, Massachusetts, USA). This approach is inherently stochastic and flexible for accommodating individual-level contact structure and spatial movement patterns. Characteristics (e.g., reproductive, disease, age, location, etc) of each individual were tracked in a matrix which was updated daily. Population-level status such as numbers of new cases, new births and spatial area were extracted each day from the individual-based matrix. The main algorithm completed the following events daily: dispersal, new conceptions, new births, disease transmission, disease-induced mortality, natural mortality, and culling. The mechanics of these events are described in more detail below.

### Demographic model

Conceptions in reproductively mature females occurred continuously throughout the year with two peaks annually using an empirically-determined seasonality in conception probability (Table 1). We assumed that conception of females occurred irrespective of males. The time-varying conception probability was implemented each day by assigning all conception-ready females (reproductively mature, non-pregnant, non-lactating) a random uniform number and choosing those with a number below the conception probability for that day of the year. Conception probability could be multiplied by a scaling factor to manipulate population growth rate (Table 1). When conception was successful, there was a gestation period (115 days, Table 1) before new births occurred. Pregnant females farrowed litters of a fixed number (number depended on age, Table 1) with a male:female ratio of 1^17^. After farrowing, females were unable to conceive again until after a waiting period that represented a lactational anestrus time.

Natural death rate was modeled *via* a longevity parameter (Table 1), which was assigned at birth from an exponential distribution, which assumes that the probability of living longer was smaller than the probability of dying young. Individual were removed once their longevity was reached. We assumed no density-dependence on births^33^ or deaths but population growth rate could vary. We chose this method of modeling the population dynamics because we were interested in the effects of contact structure under a range of population growth trajectories ranging from exponential growth^38^ to decline due to culling. Examples of the spatial and demographic dynamics are in Figures S1 and S2.

### Social structure

**Figure 1:**
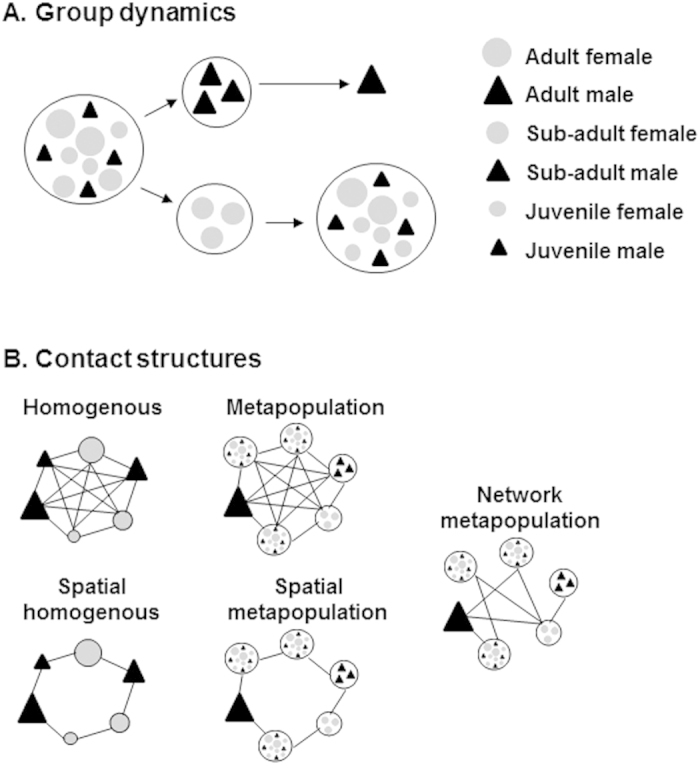
Schematic showing how group dynamics (**A**) and contact structure (**B**) were modeled. (**A**) Family groups consist of females and juveniles. Young males disperse together at reproductive maturity and eventually exist independently. Reproductively active, younger females disperse to form new family groups when a group reaches carrying capacity. (**B**) Homogenous –individuals contact other individuals with equal probability. Metapopulation – groups contact other groups with equal probability. Spatial contact structures are the same as their non-spatial counterparts but are limited to contacts within a fixed distance of their home range centroids. Network – groups only contact groups they are connected to as described by network connections. Contact structure within all groups is homogenous.

**Figure 2:**
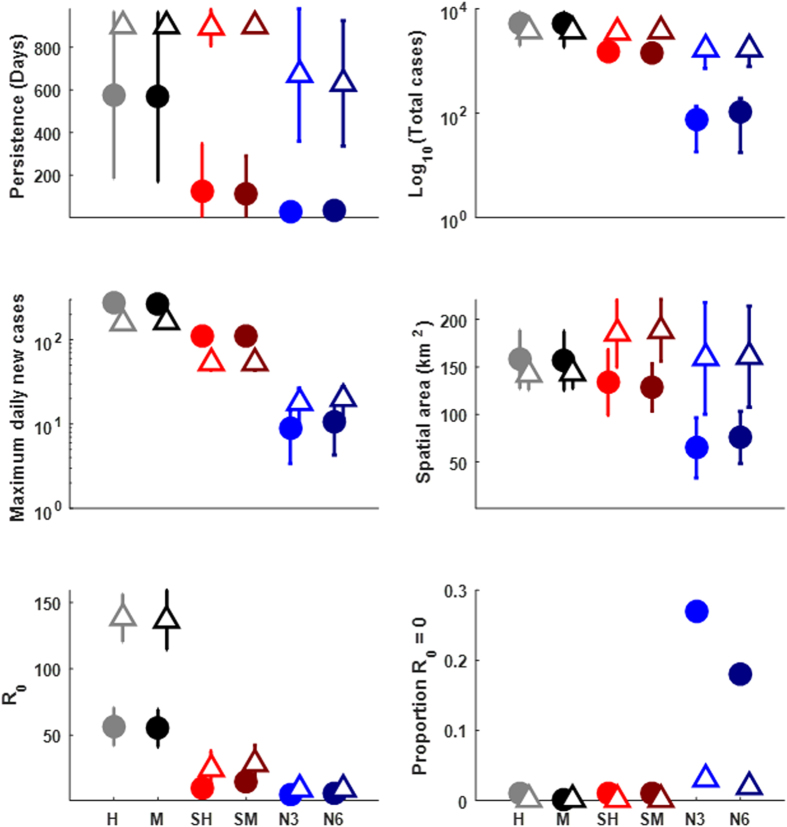
Outcome of disease dynamics for the FMDV-like (closed circles) and CSFV-like viruses (open triangles). Parameters used in sensitivity analysis were fixed at: maxK = 40, Initial density = 15, Dispersal = 3 km, within-group contact rate = 0.1, between-group contact rate = 0.1, q = 0 (density-dependent transmission). Points are the mean of 100 replicate simulations, error bars are the standard deviation of the means. Simulations were run for 4 years. A single infectious individual was seeded at 1.5 years. Parameters were as in Table 1 except that the following parameters were fixed at realistic/permissive values: scaling factor on conception probability (1 which is equivalent to an annual population growth of 120% following the index case), dispersal distance (3 km), maximum family group size (40 feral swine), initial density (15 feral swine/km^2^), within-group transmission rate (0.1/feral swine/day), between-group transmission rate (0.1/feral swine/day), and disease-induced mortality (FMDV-like virus – 0%, CSFV-like virus -50%). Network properties were mean degree 3 and global transitivity 0.76 (light blue) and mean degree 6 and global transitivity 0.74 (dark blue). Other contact structures were: homogenous (H), metapopulation (M), spatial homogenous (SH) and spatial metapopulation (SM).

**Figure 3:**
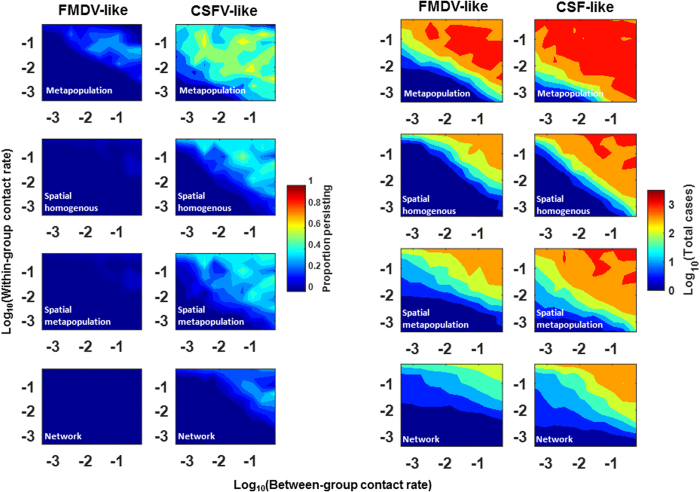
Relationship of within- and between-group transmission rates on outcome. (**A**) Heat maps of the proportion of simulations (N = 50) where infectious individuals continue to transmit after 2.5years. (**B**) The mean number of total cases in simulations where total cases were >10 (i.e., arbitrary criterion for an “outbreak”). Each column of plots are results for either the FMDV-like or CSFV-like viruses (labeled on top). Each plot shows results form a particular between-group contact structure (labeled in white in the plot). Heat map response values were averages across all sets of parameters in the sensitivity analysis. Table 1 gives the ranges of variable and fixed parameters. Network properties were mean degree 3 and global transitivity 0.76. Parameters were as in Table 1.

**Figure 4:**
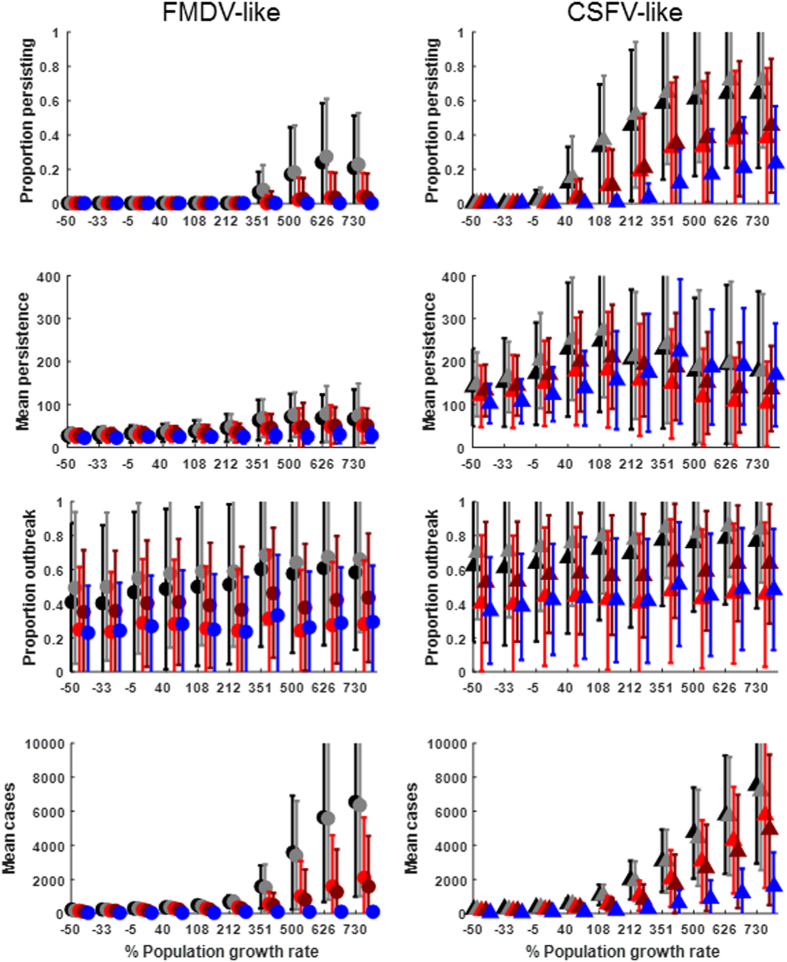
Effects of host population growth rate on disease transmission. (**A**) The proportion of simulations (N = 50) where infectious individuals continue to transmit after 2.5years. (**B**) The mean persistence in days for simulations that fade out before 2.5 years. (**C**) The proportion of simulations (N = 50) that have >10 total cases (i.e., arbitrary criterion for an “outbreak”). (**D**) The mean number of total cases for simulations that “outbreak”. X-axis represents the percent population growth in the 2.5 years following introduction of a virus. Left column of plots are for FMDV-like parameters; right column are for CSFV-like parameters. Error bars are standard deviations of the mean for all simulations with X-axis parameter values. Table 1 gives the ranges of variable and fixed parameters. Colors correspond to different between-group contact structures: Homogenous (black), Metapopulation (grey), Spatial homogenous (red), Spatial metapopulation (dark red), Network with mean degree 3 and global transitivity 0.76 (blue). Parameters were as in Table 1.

**Figure 5:**
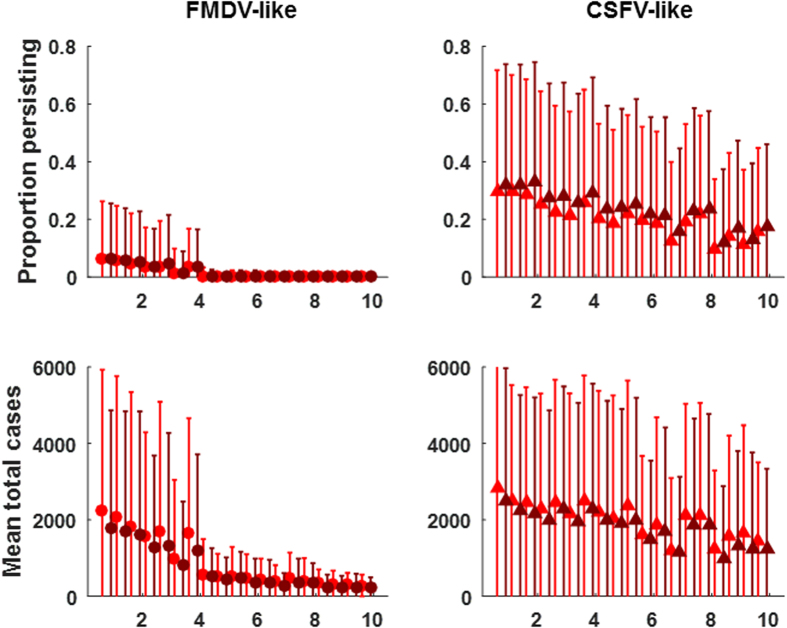
Effects of dispersal distance on outcome under spatial contact structure. (**A**) The proportion of simulations (N = 50) where infectious individuals continue to transmit after 2.5years. (**B**) The mean number of total cases for simulations that “outbreak”. X-axis represents distance of dispersal for both males at reproductive maturity and females that disperse when family groups reach carrying capacity. Left column of plots are for FMDV-like parameters; right column are for CSFV-like parameters. Error bars are standard deviations of the mean for all simulations with X-axis parameter values. Table 1 gives the ranges of variable and fixed parameters. Colors correspond to different between-group contact structures: Spatial homogenous (red), Spatial metapopulation (dark red). Parameters were as in Table 1.

**Figure 6:**
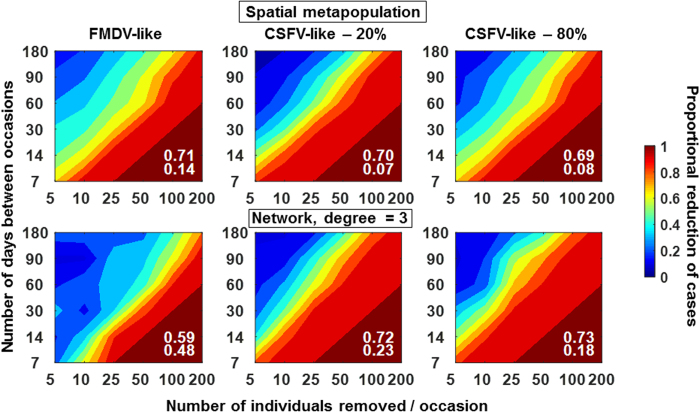
Pre-outbreak population management. Number of individuals culled per event (X-axis) corresponded to 0.9, 1.7, 4.4, 8.7, 17.4, 35.8% of the population during the initial culling occasions. Top: spatial metapopulation contact structure, Bottom: Network with degree = 3. Colors indicate the proportion of reduction in cases due to culling; i.e., (mean without culling-mean with culling)/mean without culling. Data in the plots were derived from the mean of 100 replicate simulations for each set of culling conditions. Numbers in white show the mean proportional reduction across all culling conditions in the plot (top) and the mean standard deviation across all standard deviations in the plot (bottom). Standard deviation plots are shown in Figure S3. Parameters were as in Table 1 except that the following parameters were fixed at realistic/permissive values: scaling factor on CP (1 which is equivalent to an annual population growth of 120% following the index case in the absence of culling), dispersal distance (3 km), maximum family group size (40 feral swine), initial density (15 feral swine/km^2^), within-group transmission rate (0.1/feral swine/day), between-group transmission rate (0.1/feral swine/day), and disease-induced mortality (FMDV-like virus –0%, CSFV-like virus −20% & 80%).

**Figure 7:**
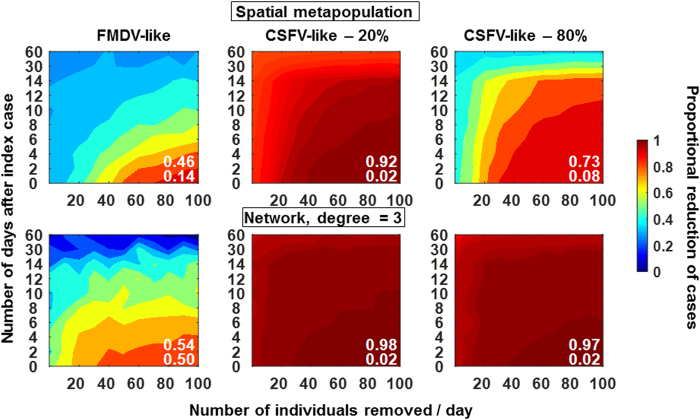
Post-outbreak response. Number of individuals culled per day (X-axis) ranged from 0.5–10% of the population at the time of disease introduction. Y-axis indicates the day daily culling begins after disease introduction. Top: spatial metapopulation contact structure, Bottom: Network with degree = 3. Colors indicate the proportion of reduction in cases due to culling; i.e., (mean without culling-mean with culling)/mean without culling. Data in the plots were derived from the mean of 100 replicate simulations for each set of culling conditions. Numbers in white show the mean proportional reduction across all culling conditions in the plot (top) and the mean standard deviation across all standard deviations in the plot (bottom). Standard deviation plots are shown in Figure S6. Parameters were as in Table 1 except that the following parameters were fixed at realistic/permissive values: scaling factor on CP (1 which is equivalent to an annual population growth of 120% following the index case in the absence of culling), dispersal distance (3 km), maximum family group size (40 feral swine), initial density (15 feral swine/km^2^), within-group transmission rate (0.1/feral swine/day), between-group transmission rate (0.1/feral swine/day), and disease-induced mortality (FMDV-like virus – 0%, CSFV-like virus −20 & 80%).

Females and young occurred in family groups (Fig. 1A). All males dispersed from natal family groups in subgroups at an age that approximated the age males begin to seek mates (5 months, Table 1, Fig. 1A). Males dispersed a second time once they became older adult males (>2 years), to become independent. We assumed that females only dispersed once group size reached maximum capacity (Fig. 1A). The group structure allowed for different disease transmission rates within and between family groups. Dispersal allowed for time-varying differences in the contact structure.

### Disease transmission

Disease states of hosts included susceptible (S), exposed but not infectious (E), infectious (I) or recovered (R). We used incubation and infectious periods that were similar to strains of FMDV^22^ and CSFV^34^,^39^. Incubation and infectious periods for each exposed individual were chosen from a Poisson distribution truncated at 1, introducing individual-level heterogeneity. We assumed no disease-induced mortality for the FMDV-like scenarios^22^, which is not realistic for juveniles with some strains^36^. For CSFV-like viruses, we used a range of disease-induced mortality conditions reflecting the situation in nature. Thus, the FMDV-like case represented a situation with very high transmission potential which we compared to the transmission potential of a CSFV-like virus over a range of disease-induced mortality effects. We did not explicitly model airborne spread, environmental transmission or vertical transmission for either virus. The force of infection was: (π_w_+π_b_)IS, where π_w_ is the within-group transmission probability, π_b_ is the between-group transmission probability, I is the number of infectious individuals and S is the number of susceptible individuals (i.e., both within and between-group transmission rates were density dependent). New exposures occurred when susceptible individuals within the contact zone of infectious individuals were selected based on the transmission probability criterion (mechanics similar to the conception process described above).

We compared 5 different contact structures: homogenous mixing, metapopulation, spatial homogenous, spatial metapopulation, and network (Fig. 1B). Thus, the number of potential contacts between individuals could vary depending on their home range centroids, group membership and/or network connections. We assumed that mixing within groups was homogenous; i.e., heterogeneous contact structure operated at the between-group level. For the spatial contact structures, groups only contacted groups within a fixed distance, making no contact with groups outside this radius. For network structures, between-group connections were determined by simulating random networks with properties (i.e., degree, transitivity, clustering) as determined in^13^. The previous work found that feral swine social networks had low mean degree (e.g., on average a group of feral swine was connected to ≤3 other groups for direct contact and ~6 groups by indirect contact through the environment) and high global transitivity (e.g. on average the proportion of closed triangles to all triangles was ~0.76). These properties were used to simulate random networks using the R package “igraph” and by adaptation of an algorithm developed by^40^. Note that the number of between-group connections for a given individual could vary over their lifetime due to dispersal.

### Sensitivity analyses

For seven parameters (scaling parameter on conception probability, initial density, mean dispersal distance, within-group contact rate, between-group contact rate, maximum group size, and disease-induced mortality; Table 1), sparse or no data were available and thus we presented results over a wide range of values to adequately represent our uncertainty. A latin-hypercube design (e.g.^41^) was used to draw 5000 sets of the uncertain parameter values. Fifty replicate simulations were run for each set of parameters. Each run allowed for a population growth period of 1.5 years before introduction of a single infectious individual in a randomly selected host. Simulations were initiated with 500 feral swine; population size ranging from 280 to 1337 at the time of virus introduction (depending on the scaling factor on conception probability). Simulations ran for 2.5 years after the index case. Annual population growth rates following virus introduction ranged from −20 to 290%. The following outputs were calculated for each unique set of parameters: 1) *Persistence probability* - the proportion of runs that contained infectious individuals after 2.5 years, 2) *Outbreak probability* - the proportion of runs with >10 total cases (“outbreak”), 3) Mean persistence – for runs that fade out, the average persistence time, and 4) Mean total cases – for runs with >10 cases, the average total cases. We used logistic models with all 6 (FMDV-like virus) or 7 (CSFV-like virus) parameters (standardized) as main effects to estimate the relative strength of each parameter on outputs 1 and 2. For outputs 3 and 4, we modeled log-transformed persistence time and total cases for all 50 runs with each unique parameter set (5000 × 50) using linear mixed models with all 6 (FMDV-like virus FMDV) or 7 (CSFV-like virus) parameters (standardized) as main effects and the unique parameter set as a random effect. Again, fixed effects were standardized to estimate the relative strength of effects of each parameter.

### Pre-outbreak management

To assess effects of current management on potential to reduce disease transmission, we examined effects of different management strategies on total cases using 2 contact structures that are likely most realistic: spatial metapopulation and network (degree = 3). For the spatial metapopulation we compared a strategy where pigs are culled at random in space versus choosing all individuals close in space. For networks, we compared culling at random to choosing the highest-degree nodes and their neighbors. We examined a range of values for number culled per event and time between events. We calculated the mean from 100 simulations for each set of parameters. We expressed the result as the proportional reduction in total cases due to management; i.e., (mean without culling-mean with culling)/mean without culling. Parameters were as in Table 1 except that the following parameters were fixed at realistic/permissive values: scaling factor on CP (1 which is equivalent to an annual population growth of X), dispersal distance (3 km), maximum group size (40 feral swine), initial density (15 feral swine/km^2^), within-group transmission rate (0.1/feral swine/day), between-group transmission rate (0.1/feral swine/day), and disease-induced mortality (CSFV-like virus only, 20 or 80%).

### Post-outbreak response

In cases of disease emergence, resources are focused in areas where the disease is detected. Given that surveillance is ongoing and the location of at least some disease-positive individuals is known, we examined the effects of initial response time on total cases. For this, we assumed that a fixed number of individuals most closely located to a randomly selected disease-positive case, were removed daily from the population. We varied the number removed per day and the day post-index case that the response began. As for the pre-outbreak management, we used means from 100 replicate simulations to calculate the proportional reduction in total cases due to response. Parameters were as in the pre-outbreak management simulations.

## Additional Information

**How to cite this article**: Pepin, K. M. and VerCauteren, K. C. Disease-emergence dynamics and control in a socially-structured wildlife species. *Sci. Rep.* **6**, 25150; doi: 10.1038/srep25150 (2016).

## Supplementary Material

Supplementary Information

## Figures and Tables

**Table 1:** Description of parameters.

	Values	References
**Demographic**		
Longevity (number of days before natural death occurs)	~EXP(λ), λ = 2 years	17,42 (Table 6, pg 173)
Daily conception probability (CP) (by age class of females)	Daily values grouped by calendar month; patterns included two weak birth pulses; annual ranges by age class were: 0.00044–0.0071 (<1 year) 0.0022–0.036 (1–3 years) 0.0044–0.071 (>3 years)	Estimated from: 17 (Fig. 1 pg 67)
Scaling factor on CP (allows annual % population growth to range from X to Y)	0.01–10	
Litter size (number of viable offspring per litter)	Fixed by age class 2 piglets (<1 year) 5 piglets (1–3 years) 7 piglets (>3 years)	43, 44, 45
Age at reproductive maturity (females only) (minimum age at which females may conceive)	6 months	46,47
Minimum time between farrowing and conception	3 months	48
Gestation time	115 days	49
Age of male dispersal from group	5 months	50,51
Age that male groups dissolve and males become independent	2 years	expert opinion
Dispersal distance (DD) (both males and females)	0.1–15 km	52
Maximum family group size (K) (carrying capacity for family groups; at carrying capacity some females will disperse and form new family groups)	5–50 feral swine	expert opinion
Initial density (ID)	1–50 feral swine/km^2^	17 (Table 4 pg 169–172)
Initial age distribution (proportion of individuals in each age class at the outset of simulations)	0–1: 56.5%; 1–2: 16.2%; 2–3: 11.1%; 3–4: 7.5%; 4–5: 4.3%; 5–6: 2.4%; >6: 1.9%	17 (Table 3, pg 168)
**Disease**		
Incubation period (FMDV-like)	~POI(λ); λ = 2 days	22
Infectious period (FMDV-like)	~POI(λ); λ = 8 days	22
Disease-induced mortality (DIM) (FMDV-like)	0	22
Incubation period (CSFV-like)	~POI(λ); λ = 8 days	34,38
Infectious period (CSFV-like)	~POI(λ); λ = 42 days	34,38
Disease-induced mortality (DIM) (CSFV-like)	0–100%	34,38
Within-group transmission probability (π_w_)	0.0003–0.99/infected feral swine/day	NA
Between-group transmission probability (π_b_)	0.0003–0.99/infected feral swine/day	NA
Spatial cutoff (limiter for contact in spatial models)	2 km	13

^*^Underlined parameters are those that were varied using a Latin-hypercube design (ranges of values are indicated). These were fixed among individuals within a simulation. ^*^Parameters with distributions were random variables chosen from their specified distribution. These were variable among individuals within a simulation. ^*^Other parameters were fixed among individuals within the same simulation and across all simulations.
