# Supplementary material for: Disease-emergence dynamics and control in a socially-structured wildlife species
Source: Sci Rep. 2016 Apr 26;6:25150. doi: 10.1038/srep25150 (PMC4844964; doi:10.1038/srep25150)
Supplement: Supplementary Information [file srep25150-s1.pdf]

Disease-emergence dynamics and control in a socially-structured wildlife species

Kim M. Pepin and Kurt C. VerCauteren

Supplementary Figures

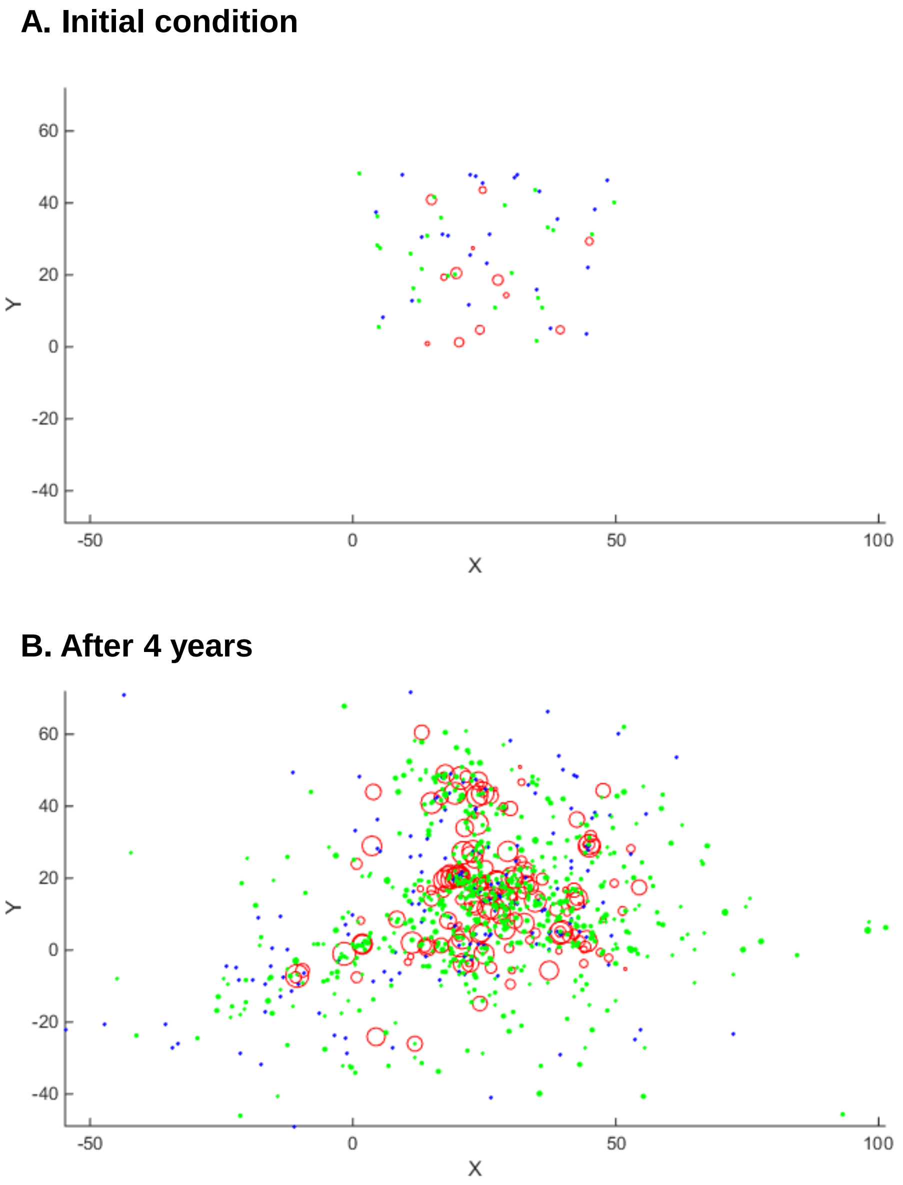

Figure S1. Example of spatial spread during the course of a 4-year simulation. Size of points is correlated to the number of individuals in a group. Red: sounders. Green: young male groups. Blue: Individual adult males.

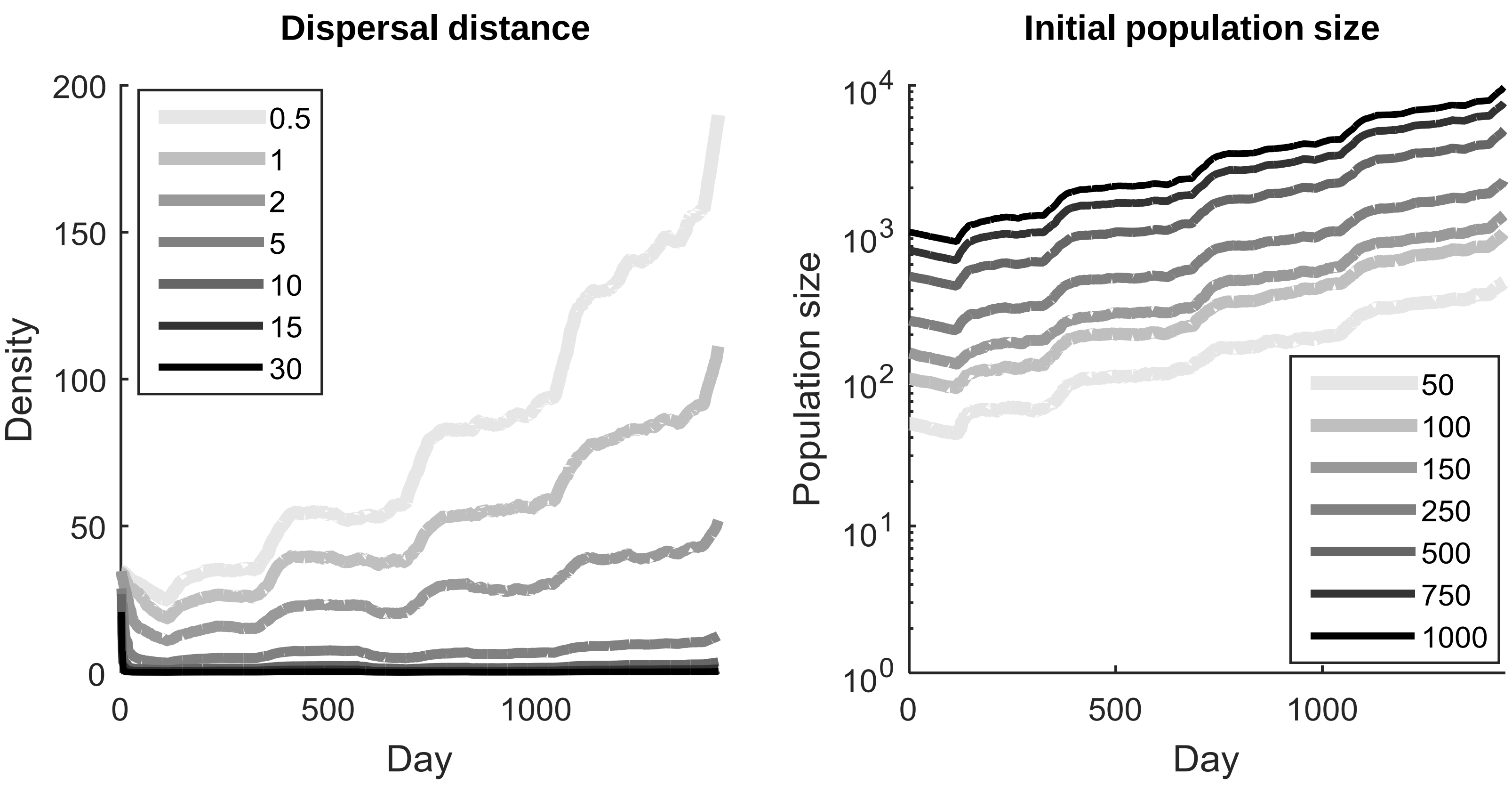

Figure S2. Example of demographic dynamics. Parameters in main text were: dispersal = 3 km, initial population size = 500. Population density is relatively low and stable (after the burnin period) for dispersal distances < 5 km.
After 4 years a population of 500 grows to 5000 individuals.

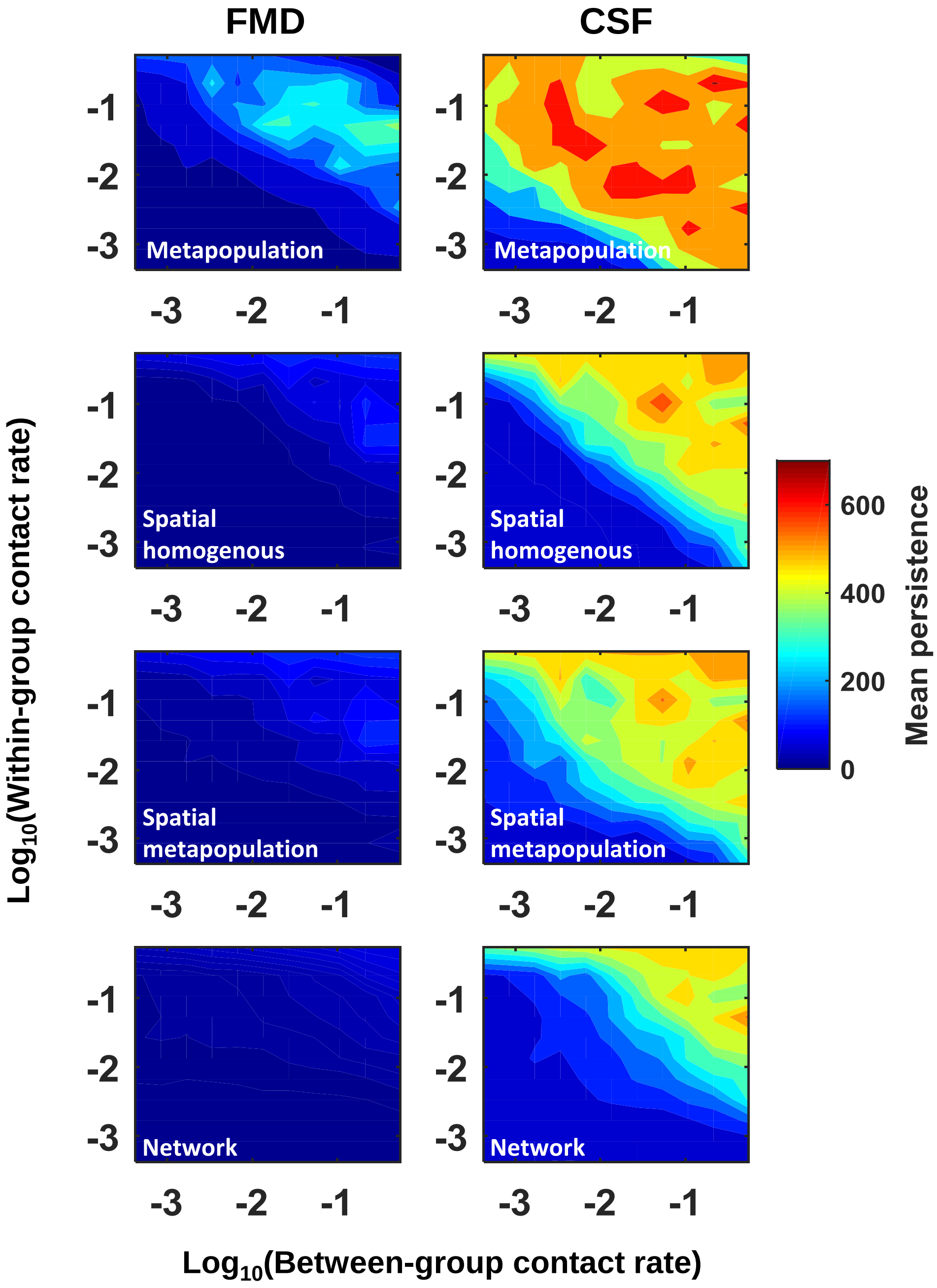

Figure S3. Sensitivity of mean persistence to within and between-group contact rates for outbreaks that fade out before the end of simulations (2.5 years). Each plot represents results from a different contact structure (as indicated above the plot).

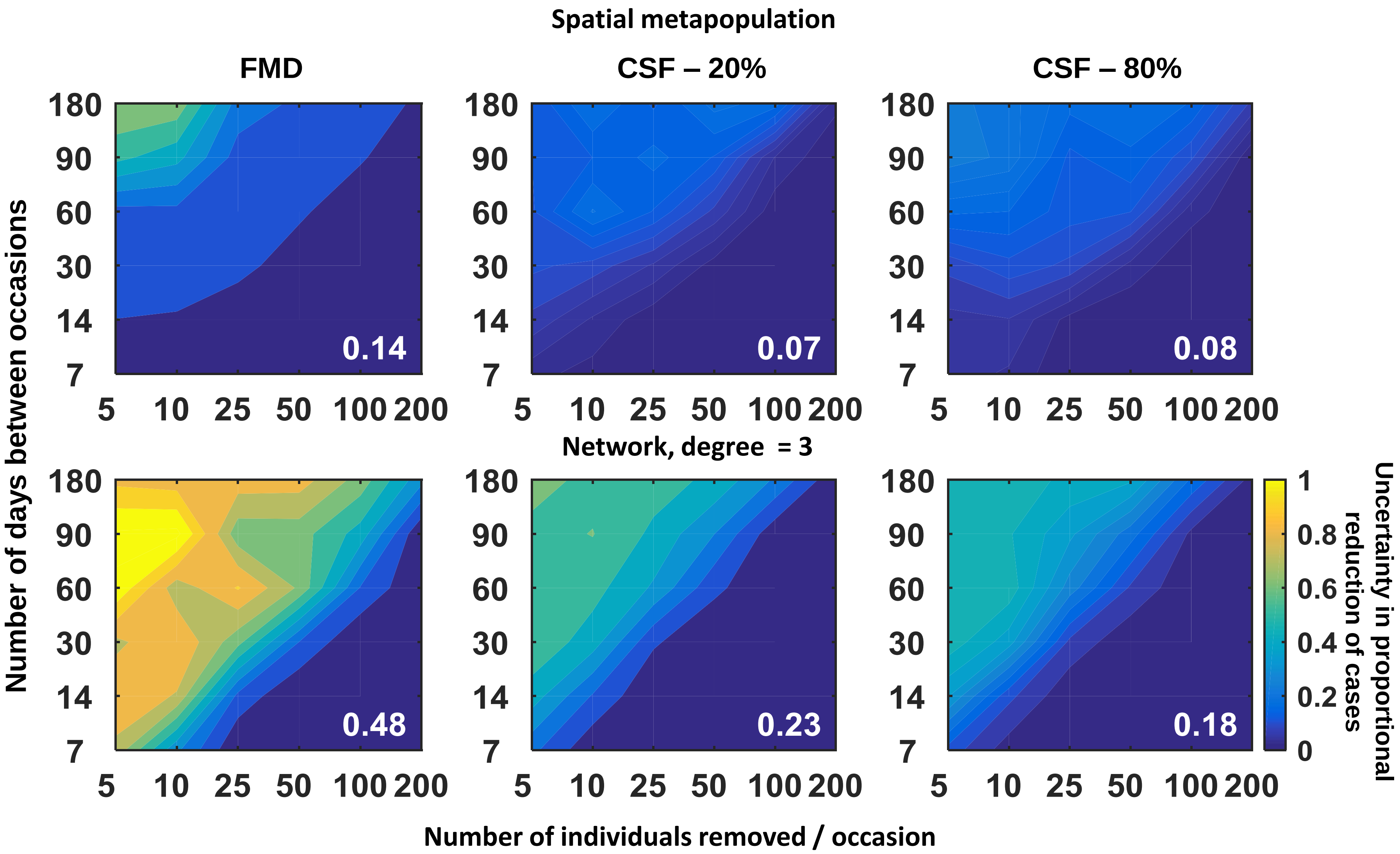

Figure S4. Uncertainty for pre-outbreak population management. Same as Figure 6 but heat maps are for standard deviations of 100 replicate simulations for each set of parameters. Numbers in white are the average standard deviations across all values in the plot.

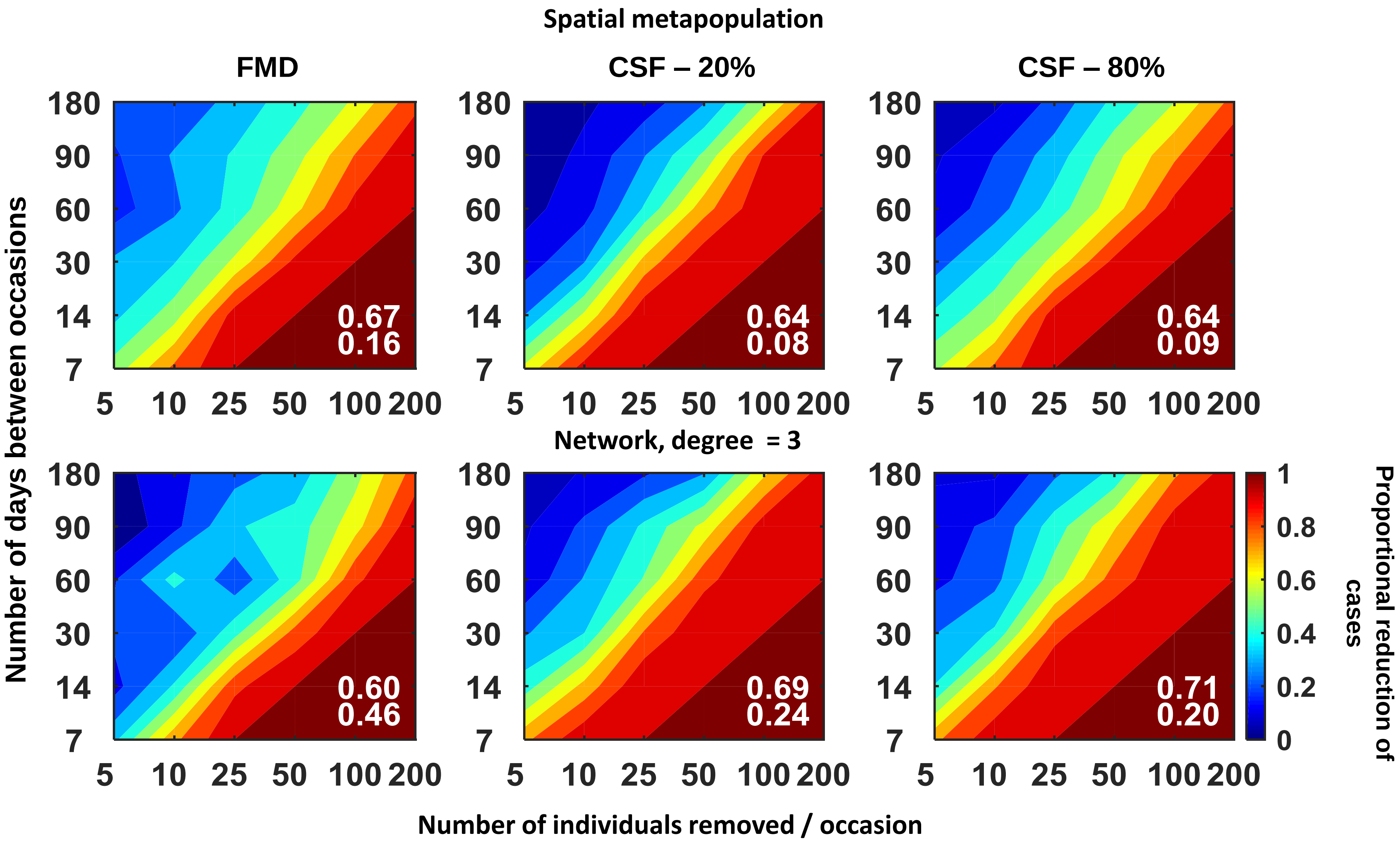

Figure S5. Pre-outbreak population management. Same as Figure 6 but individuals were selected at random across the landscape for culling.

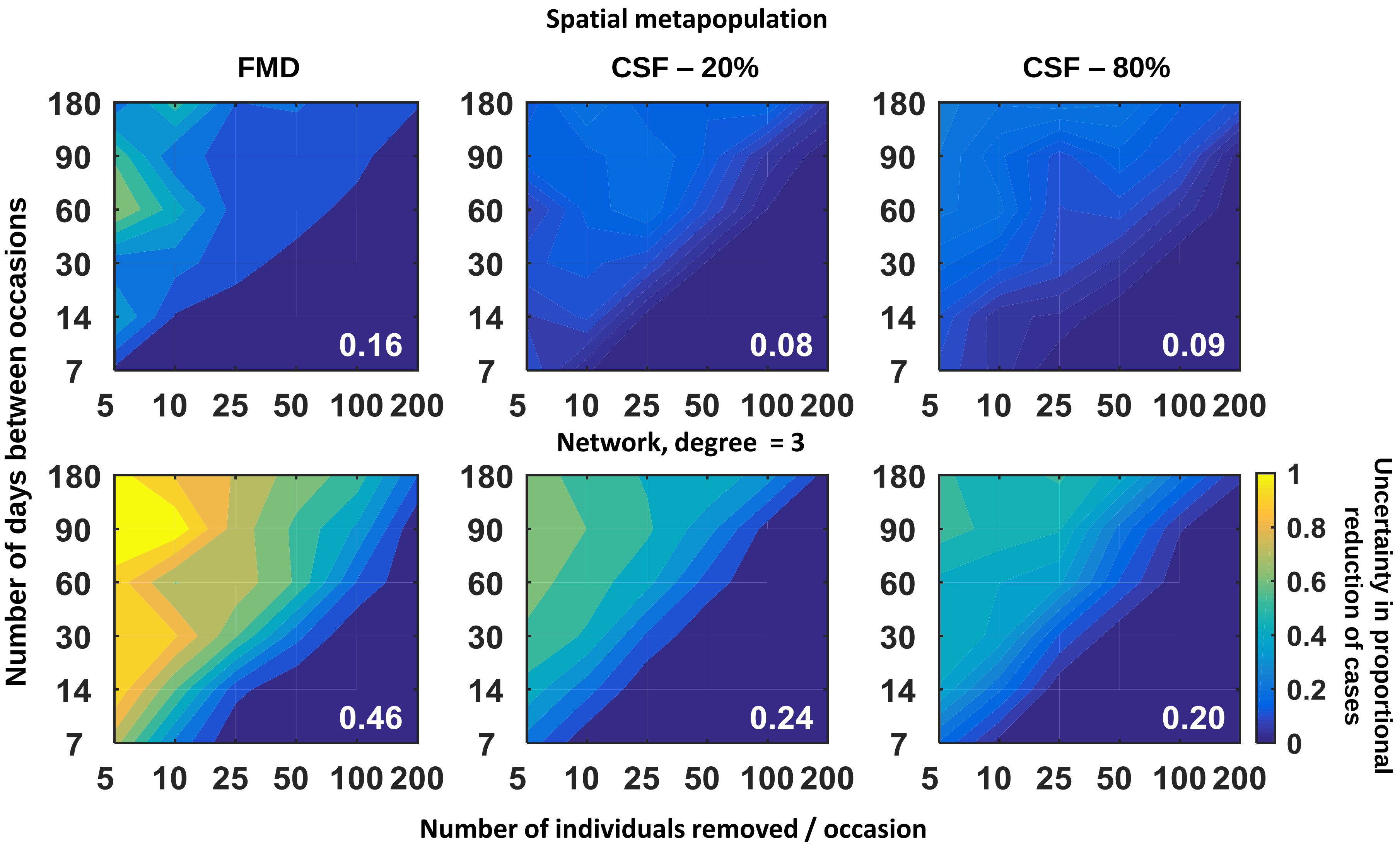

Figure S6. Uncertainty for pre-outbreak population management. Same as Figure S5 but heat maps are for standard deviations of 100 replicate simulations for each set of parameters. Numbers in white are the average standard deviations across all values in the plot.

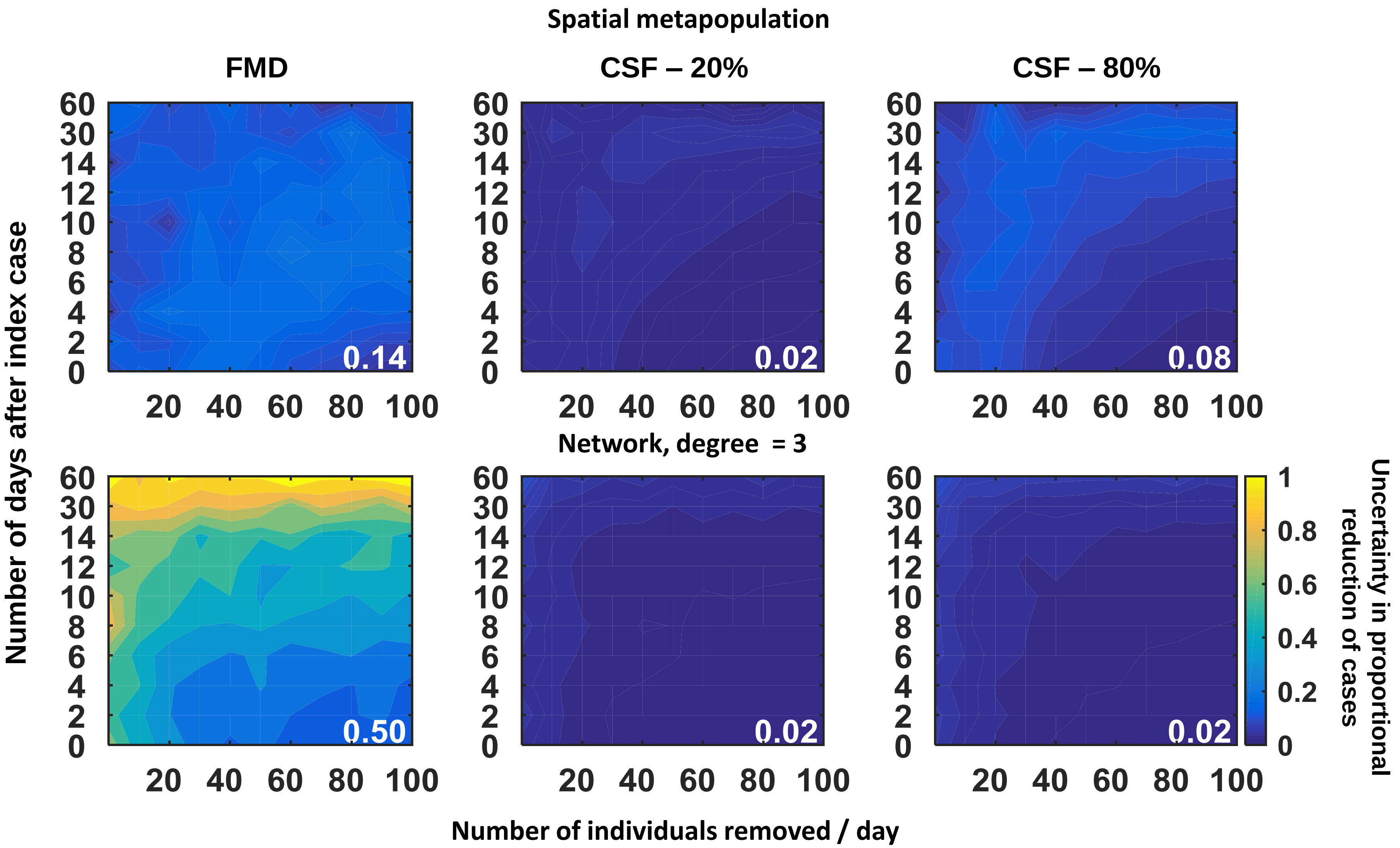

Figure S7. Uncertainty for post-outbreak response. Same as Figure 7 but heat maps are for standard deviations of 100 replicate simulations for each set of parameters. Numbers in white are the average standard deviations across all values in the plot.
